# Comparison of Transcatheter Aortic Valve Implantation *versus* Surgical Aortic Valve Replacement to Improve Quality of Life in Patients >70 Years of Age with Severe Aortic Stenosis

**DOI:** 10.5935/1678-9741.20150092

**Published:** 2016

**Authors:** Cemal Kocaaslan, Bülend Ketenci, Mehmet Yılmaz, Tamer Kehlibar, Mehmet Erdem Memetoğlu, Gökhan Ertaş, Mehmet Eren, Mahmut Murat Demirtaş

**Affiliations:** 1 Dr.Siyami Ersek Cardiovascular and Thoracic Surgery Hospital, İstanbul, Turkey.

**Keywords:** Aortic Valve, Surgery, Aortic Valve Stenosis, Quality of Life, Heart Valve Prosthesis Implantation

## Abstract

**Objective:**

Transcatheter aortic valve implantation has recently been used in the treatment of severe aortic valve stenosis, particularly in patients with high mortality and morbidity rates for open surgery. The purpose of this study was to compare quality of life in patients over 70 years of age undergoing surgical or transcatheter aortic valve implantation, before the procedure and in the early post-procedural period.

**Methods:**

Seventy-nine patients were included in the study, 38 (48.1%) male and 41 (51.9%) female. Mean age of patients was 74.3±5.2 (70-91) years. The surgical aortic valve replacement group consisted of 51 (64.6%) patients and the transcatheter aortic valve replacement group of 28 (35.4%). Quality of life data before the procedure and at the 3^rd^ month postoperatively in patients aged 70 years and older undergoing surgical or transcatheter aortic valve implantation were assessed using the 36-item Short Form Health Survey form.

**Results:**

Positive increases in physical task difficulty (13.2±9.8 *vs.* 5.1±7.3) (*P*=0.001), emotional task difficulty (14.4±11.9 *vs.* 8.5±6.4) (*P*=0.035), and mental health (0.4±10.4 *vs.* 9.6±15.1) (*P*=0.001; *P*<0.01) scores in patients undergoing transcatheter aortic valve replacement were significantly higher compared to the surgical aortic valve replacement group. No statistically significant difference was determined between the groups in terms of pain, vitality, social function, physical function or general health scores in the preoperative and postoperative periods.

**Conclusion:**

The positive increase in quality of life parameters in the transcatheter aortic valve implantation group at the 3^rd^ month postoperatively was significantly higher compared to the surgical aortic valve replacement group.

**Table:** 

**Abbreviations, acronyms & symbols**	
AS	= Aortic stenosis
AVR	= Aortic valve replacement
DM	= Diabetes mellitus
EuroSCORE	= European System for Cardiac Operative Risk Evaluation
NYHA	= New York Heart Association
PARTNER	= Placement of Aortic Transcatheter Valve
SF-36	= Short Form 36
TA	= Transapical
TAVI	= Transcatheter aortic valve implantation
TF	= Transfemoral

## INTRODUCTION

Aortic stenosis (AS) is a common valve disease and if left untreated, the prognosis of severe AS is poor. A surgical approach involving open heart surgery with low levels of mortality and acceptable long-term morbidity levels has been successfully applied in severe AS. Nonetheless, the risk of perioperative morbidity and mortality increases in elderly patients or those with accompanying diseases and they may be regarded as inoperable.

Transcatheter aortic valve implantation (TAVI) was initially introduced by Cribier and colleagues and, currently, TAVI represents a valid therapeutic option for patients with severe aortic stenosis who are inoperable or are at very high risk for conventional surgery^[1,2]^. TAVI is performed using one of two different approaches: the retrograde transfemoral (TF) approach via the femoral artery or the antegrade transapical (TA)^[3]^.

Quality of life is a subjective perception regarding an individual's state of well-being, depending on sociocultural structures^[4]^. Multidimensional evaluation of quality of life in terms of physical, psychological and social functioning is reported to be a good marker of an individual's health status^[5]^.

Various scales can be employed in the evaluation of patients' quality of life and health outcomes. One of the most widely used is the Medical Outcome Study Short Form-36 (SF-36). This is an easily applied test that provides important information about quality of life assessment in patients undergoing open heart surgery and percutaneous cardiac procedures^[6]^. SF-36 consists of 36 items, including 8 separate health-related dimensions. The form is divided into the following domains: physical function (10 items), social function (2 items), task restrictions due to physical problems (4 items), task restrictions due to emotional problems (3 items), mental health (5 items), vitality (4 items), pain (2 items), and general health (5 items). The items in SF-36 inquire into positive or negative status concerning health and are assessed based on the preceding 4 weeks. Item scores are coded for each dimension and converted into a scale from 0 (worst health status) to 100 (best health status). There have been limited data concerning the contribution to patients' quality of life of TAVI.

The purpose of this prospective study was to use the SF-36 test to assess quality of life in patients over 70 years of age undergoing aortic valve replacement (AVR) or TAVI due to AS in our hospital, before the procedure and at the 3^rd^ postoperative month.

## METHODS

AVR was planned on 79 patients aged over 70 diagnosed with advanced aortic stenosis between January and June 2014. Approval for the study was granted by the hospital's research ethics committee. Participants were informed about the study and signed an informed consent form.

All patients were assessed in terms of aortic structure, porcelain aorta, penetrating ulcer, and suitability of iliac arteries for catheter using computed tomography angiography. Patients were investigated in terms of age, sex, hypertension, diabetes mellitus (DM) and history of cigarette use, and parameters obtained from tests, analyses and examinations were scored using the European System for Cardiac Operative Risk Evaluation (EuroSCORE) risk scoring system. A hospital council, comprised of specialist cardiologists, anesthesiologists and radiologists, discussed every patient's case and decided on the type of procedure to be performed.

Only patients with advanced AS aged 70 or over were enrolled in the study. Patients with other cardiac procedures in the same session besides AVR were excluded.

Severe AS was defined by the criteria used in the Placement of Aortic Transcatheter Valve (PARTNER) Trial^[7]^: an aortic valve area of <0.8 cm2 (or aortic valve area index <0.5 cm^2^/m^2)^, a mean aortic gradient of >40 mmHg, or a peak aortic jet velocity of >4 m/s. All patients had a New York Heart Association (NYHA) functional class ≥2. The exclusion criteria included recent acute myocardial infarction (≤1 month), recent stroke or transient ischemic attack (within 6 months), congenital bicuspid aortic valves, preexisting prosthetic heart valve, severe ventricular dysfunction (left ventricular ejection fraction <20%), renal insufficiency (creatinine >3 mg/dL), and life expectancy of <12 months.

### Procedure

Prior to both procedures, patients were informed about how the procedure would be performed and its possible risks. AVR using St. Jude Medical® Mechanical heart valve (St. Jude Medical Inc.; Minneapolis, MN, USA) and TAVI using the Edwards Sapien valve (Edwards Lifesciences, Irvine, CA, USA) were performed under general anesthesia and using standard procedures. Patients were taken to the intensive care unit after both procedures and remained there for at least one night. Patients with no complications and who improved on the first day were discharged.

### Quality of life assessment

The SF-36 was used in the measurement and evaluation of quality of life. The SF-36 was administered to 51 patients scheduled for AVR and 28 patients scheduled for TAVI one day before and 3 months after the procedure, and quality of life status was recorded. Patients completed the form either alone or with the help of relatives.

### Statistical analysis

Statistical analysis was performed on IBM SPSS Statistics 22 (IBM SPSS, Turkey) software. The Shapiro Wilks test was used to determine normal distribution of data. Descriptive statistical techniques were used to analyze the study data (mean plus standard deviation). In addition, the Mann Whitney U test was used to compare parameters between the two groups and the Wilcoxon signed test for intragroup was used for pre- and postoperative comparisons. Chi square test, Fisher's exact chi square test and Yates continuity correction were used in the comparison of qualitative data. A value of *P*<0.05 was considered statistically significant.

## RESULTS

Seventy-nine patients were enrolled in the study, 38 (48.1%) of them were male and 41 (51.9%) were female. Mean age of patients was 74.3±5.2 years (70-91). The AVR group consisted of 51 (64.6%) patients and the TAVI group of 28 (35.4%). In-hospital mortality occurred in four patients in the AVR group and in one in the TAVI group. A further one patient in the AVR group and 3 in the TAVI group died in the second month after discharge. One patient in the AVR group was still being monitored in the chronic intensive care unit after 3 months.

Mean ages were 79.6±5.7 years in the TAVI group and 71.4±1.2 in the AVR group (*P*=0.001). Mean EuroSCORE values were 9.75±1.2 in the TAVI group and 5.65±0.8 in the AVR group (*P*=0.001). No significant differences were determined between the two groups in terms of demographic findings other than age and EuroSCORE (*P*>0.05). (Table 1)

**Table 1:** Patients’ demographic and clinical data according to groups.

		AVR	TAVI	*P *
Age _(mean±SD)_		71.43±1.25 (71)	79.64±5.72 (81)	0.001**
EF _(mean±SD)_		46.76±4.88 (45)	46.96±4.97 (45)	0.880
EuroSCORE _(mean±SD)_		5.65±0.82 (5)	9.75±1.27 (9)	0.001**
Gender _n=_number,%__	Male	n=27 (52.9%)	n=11 (39.3%)	0.354
	Female	n=24 (47.1%)	n=17 (60.7%)	
DM _number,%_	(+)	n=36 (70.6%)	n=21 (75%)	0.876
	(-)	n=15 (29.4%)	n=7 (25%)	
HT _number,%_	(+)	n=45 (88.2%)	n=25 (89.3%)	1,000
	(-)	n=6 (11.8%)	n=3 (10.7%)	
Smoking _number,%_	(+)	n=30 (58.8%)	n=14 (50%)	0.604
	(-)	n=21 (41.2%)	n=14 (50%)	
NYHA _number,%_	II	n=7 (13.7%)	n=5 (17.9%)	0.703
	III	n=39 (76.5%)	n=19 (67.9%)	
	IV	n=5 (9.8%)	n=4 (14.3%)	

AVR=aortic valve replacement; TAVI=transcatheter aortic valve implantation; EF=ejection fraction; HT=hypertension; DM=Diabetes Mellitus; NYHA=New York Heart Association;

SD=standard deviation **P<0.01

**Fig. 1:**
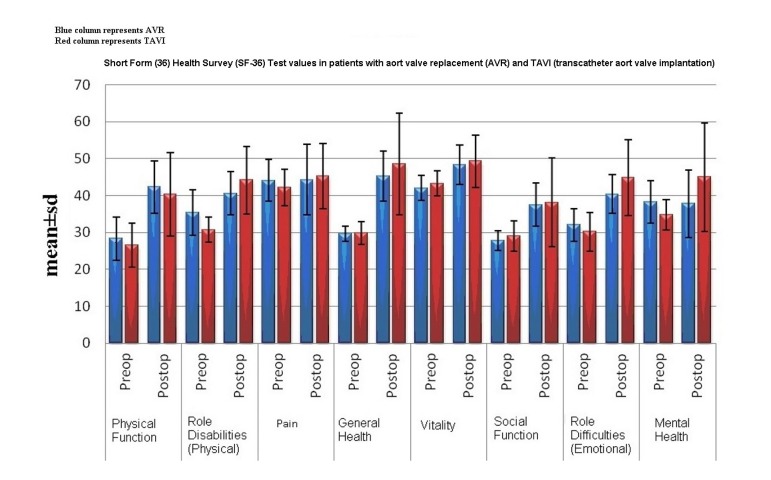
Short Form (36) Health Survey (SF-36) Test values in patients with aortic valve replacement (AVR) and TAVI (transcatheter aortic valve implantation); blue column represents AVR, red column represents TAVI.

Although preoperative physical task difficulty scores in the AVR group were significantly higher than those in the TAVI group (35.3±6.1 *vs.* 30.7±3.4; *P*=0.001), no statistically significant difference was observed between postoperative physical task difficulty scores. The level of positive change in physical task difficulty scores in the TAVI group was significantly higher than that in the AVR group (13.2±9.8 *vs.* 5.1±7.3; *P*=0.001) (Table 2, Figure 1).

**Table 2:** Evaluation of the SF-36 scores according to groups.

		AVR	TAVI	*P *
		Mean±SD (Median)	Mean±SD (Median)	
Physical Function	Preop	28.29±5.77 (27.8)	26.53±5.87 (25.7)	0.126
	Postop	42.3±7.07 (38.3)	40.3±11.34 (42.5)	0.783
	Dif	13.55±7.74 (12.6)	13.64±10.91 (14.7)	0.933
	^2^ *P*	0.001*	0.001**	
Role Disabilities (Physical)	Preop	35.31±6.14 (35)	30.75±3.48 (28)	0.001**
	Postop	40.59±5.91 (42,1)	44.16±9.18 (42,1)	0.060
	Dif	5.1±7.34 (7)	13.24±9.82 (14.1)	0.001**
	^2^ *P*	0.001**	0.001**	
Pain	Preop	44.11±5.59 (46.5)	42.12±4.96 (42.2)	0.156
	Postop	44.27±9.54 (46.5)	45.28±8.87 (46,05)	0.728
	Dif	0.28±10.94 (0)	2.82±10.87 (4.3)	0.312
	^2^ *P*	0.957	0.213	
General Health	Preop	29.59±2.07 (31.2)	29.74±3.09 (28.9)	0.852
	Postop	45.27±6.83 (43.9)	48.62±13.8 (48.55)	0.217
	Dif	15.61±7.21 (15)	19.17±14.16 (18.05)	0.176
	^2^ *P*	0.001**	0.001**	
Vitality	Preop	42.04±3.38 (44.3)	43.29±3.38 (44.3)	0.134
	Postop	48.35±5.28 (49.1)	49.27±7.19 (49.1)	0.450
	Dif	6.4±5.61 (7.1)	5.65±7.42 (4.8)	0.562
	^2^ *P*	0.001**	0.002**	
Social Function	Preop	27.78±2.68 (30)	29.04±4.17 (30)	0.234
	Postop	37.51±5.85 (35.4)	38.14±12.07 (35.4)	0.955
	Dif	9.58±6.63 (10.8)	9.26±12.12 (5.4)	0.578
	^2^ *P*	0.001**	0.002**	
Role Difficulties (Emotional)	Preop	32.01±4.4 (34.3)	30.14±5.27 (34.3)	0.095
	Postop	40.33±5.25 (44.8)	44.79±10.29 (44.8)	0.019*
	Dif	8.51±6.46 (10.5)	14.47±11.96 (15.8)	0.035*
	^2^ *P*	0.001**	0.001**	
Mental Health	Preop	38.27±5.85 (39.1)	34.77±4.03 (36.8)	0.001**
	Postop	37.78±9.15 (34.5)	44.96±14.77 (48.15)	0.005**
	Dif	0.42±10.41 (2.3)	9.68±15.1 (10.2)	0.001**
	^2^ *P*	0.983	0.008**	

AVR=aortic valve replacement; TAVI=transcatheter aortic valve implantation; Dif=difference; Preop=preoperative;

Postop=postoperative; SD=standard deviation

* P<0.05

** P<0.01

There was no difference between AVR and TAVI in terms of emotional task restriction in the preoperative period, but the level of positive change in the TAVI group was significantly higher than that in the AVR group (14.4±11.9 *vs.* 8.5±6.4; *P*=0.035) (Table 2, Figure 1).

Preoperative mental health in the AVR group was significantly higher compared to the TAVI group (38.2±5.8 *vs.* 34.7±4; *P*=0.001). However, the level of positive change in mental health scores in the postoperative period in the TAVI group was significantly higher compared to the AVR group (0.4±10.4 *vs.* 9.6±15.1; *P*=0.001; *P*<0.01) (Table 2, Figure 1).

## DISCUSSION

The positive improvement in this study of patients with advanced AS aged over 70 in post-procedural physical and emotional task restriction scores in the TAVI group was higher than that in the AVR group. Although preoperative mental health scores in the AVR group were higher than those of the TAVI group, there was a very high increase in post-procedural mental health scores in the TAVI group. No significant differences in quality of life improvements were determined between the two groups in the other parameters, including pre- and postoperative pain, vitality, social functioning, physical functioning, and general health scores. Improvement in both disease-specific symptoms and general health has been observed following TAVI and AVR in patients with advanced AS. The effect on quality of life of procedural techniques has also been investigated in patients undergoing TAVI. Shorter recovery and higher levels of improvement in quality of life have been reported in patients undergoing transfemoral TAVI compared to those undergoing transapical TAVI requiring thoracotomy^[8]^.

Levels of improvement in quality of life in the postoperative period in patients unsuitable for transfemoral procedures and undergoing transapical were not higher than those in patients undergoing AVR. While a small thoracotomy incision is performed in the transapical approach, the absence of a positive change in quality of life that might be expected in transapical TAVI compared to classic AVR, which involves median sternotomy and cardiopulmonary bypass, may be attributed to greater length and severity of thoracotomy-related postoperative pain compared to those in sternotomy, resulting in greater restriction of the patient^[8]^.

The greatest benefit in quality of life following TAVI was observed in patients' physical functions, and the least in body pain. Bekeredjian et al.^[9]^ reported that TAVI had positive effects on quality of life in mental terms in patients aged over 80.

In our study, the greatest benefit observed in the TAVI group was in general health functions and the least was in body pain. TAVI was also observed to bestow significant positive benefits in terms of mental health.

One prospective study determined that quality of life at the 3^rd^ month following TAVI increased significantly compared to the preoperative period; it also showed an increase in patients' NYHA functional capacities^[10]^.

Age-related activity restrictions occur in patients aged 70 years and over. One study comparing preoperative and 6^th^ month postoperative quality of life using SF-36 in patients with a mean age of over 70 undergoing AVR reported positive changes at the 6^th^ month in physical functioning, social functioning, physical health-related task restriction, vitality, and health status. That same study also stated that functional capacity decreased from NYHA class 3 to class 1 in 82% of patients^[11]^.

Another study of patients at more advanced ages (80 years old or more) undergoing AVR reported a particular increase in functional capacities independently of age in the postoperative period in the great majority of patients. Significant improvements were also observed in general and mental health, social functioning, emotional task, and pain^[12]^.

In our study, significant, positive changes were determined in all parameters, apart from pain and mental health, in all the patients in the AVR group following the procedure.

No significant difference has been reported between mortality levels in the 1^st^ month and in the 1^st^ year due to cardiac or any other causes following TAVI or AVR in patients with advanced aortic stenosis and high comorbidity^[13]^.

One study comparing quality of life values following TAVI and AVR reported that although the TAVI group was generally ahead in the 1^st^ month, quality of life was generally similar between the two groups at the 6^th^ and 12^th^ months, and that the AVR group caught up with the TAVI over time^[14]^.

We think that studies performed at the 1^st^ month may not produce sound findings since this includes the time when patients undergoing AVR are still in recovery. Quality of life values have been shown to increase rapidly in patients undergoing AVR after a 2-month recovery period and with sternum stabilization. This was also confirmed in our study.

In terms of limitations, our findings are limited to the early period in patients undergoing TAVI and AVR. Additionally, SF-36 was used in the measurement and evaluation of patients' quality of life. We used this form because it is easy to apply, contains easily understood questions and, in particular, determines the degree of dependence on another person in patients' daily lives. The fact that, apart from assessment of quality of life, other tests such as hospital anxiety and depression scales were not administered represents another limitation.

Advanced AS is a mechanical problem that can severely affect the individual, both mentally and physically. Whether this mechanical problem is overcome with AVR or TAVI, the procedures in both groups allow patients' symptoms to be resolved, life expectancy to be extended and quality of life to be improved.

## CONCLUSION

The increase in quality of life parameters in the TAVI group at the end of the 3^rd^ month was greater than that in the AVR group. This may best be attributed to TAVI being a non-invasive method and there being no need for cardiopulmonary bypass during the procedure.

**Table:** 

**Authors’ roles & responsibilities**	
CK	Conception and study design; execution of operations and/or trials; analysis and/or data interpretation; writing of the manuscript or critical review of its content; final manuscript approval
BK	Writing of the manuscript or critical review of its content; final approval of the manuscript
MY	Writing of the manuscript or critical review of its content; final approval of the manuscript
TK	Final approval of the manuscript
MEM	Analysis and/or data interpretation; writing of the manuscript or critical review of its content; final approval of the manuscript
GE	Writing of the manuscript or critical review of its content; final approval of the manuscript
ME	Writing of the manuscript or critical review of its content; final approval of the manuscript
MMD	Execution of operations and/or trials; writing of the manuscript or critical review of its content; final approval of the manuscript
